# Supplementary material for: A Comprehensive Performance Evaluation of Deformable Face Tracking “In-the-Wild”
Source: Int J Comput Vis. 2017 Feb 25;126(2):198–232. doi: 10.1007/s11263-017-0999-5 (PMC6953975; doi:10.1007/s11263-017-0999-5)
Supplement: Supplementary file 1 — Supplementary material 1 (pdf 2664 KB) [file 11263_2017_999_MOESM1_ESM.pdf]

International Journal of Computer Vision manuscript No.
(will be inserted by the editor)

A Comprehensive Performance Evaluation of Deformable Face Tracking "In-the-Wild"

**Grigorios G. Chrysos · Epameinondas Antonakos* ·
Patrick Snape* · Akshay Asthana · Stefanos Zafeiriou**

Received: date / Accepted: date

1 Introduction

In the supplementary material, we have included all the curves as derived per detector/tracker. We follow the same experimental sections as in the main paper and include those additional curves.
Since for each method all 4 landmark localisation techniques are provided, there is a single unified legend for all the figures for each landmark localisation method, given in 1.

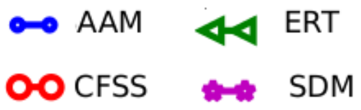

Fig. 1: The common legend for all the landmark localisation techniques used in the paper.

Additionally we have prepared a video for two indicative experiments, illustrating the visual comparison of the top 5 performing methods in each category. The links are presented in the Table 1.

---

* E. Antonakos and P. Snape contributed equally and have joint second authorship.

G. Chrysos · E. Antonakos · P. Snape · S. Zafeiriou
Department of Computing, Imperial College London, 180 Queen's Gate, London SW7 2AZ, UK
E-mail: {g.chrysos, e.antonakos}@imperial.ac.uk
{p.snape, s.zafeiriou}@imperial.ac.uk

A. Asthana
Seeing Machines Ltd., Level 1, 11 Lonsdale St, Braddon, ACT, Australia, 2612
E-mail: a.asthana@seeingmachines.com

Experiment 1	`https://youtu.be/Lx5gHvErqX8`
Experiment 3	`https://youtu.be/SNr39MH3dh8`

Table 1: Links for the videos illustrating the outcomes of the top performing methods in indicative experiments.

Each image of a video is composed of the original video frame, plus a cropped result for each of the top 5 methods per category. A black frame signifies a missing detection, Fig. 2.

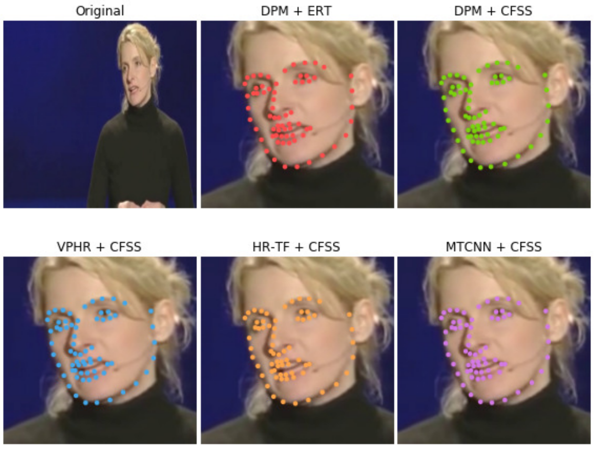

Fig. 2: Arbitrary frame from the supplementary videos.

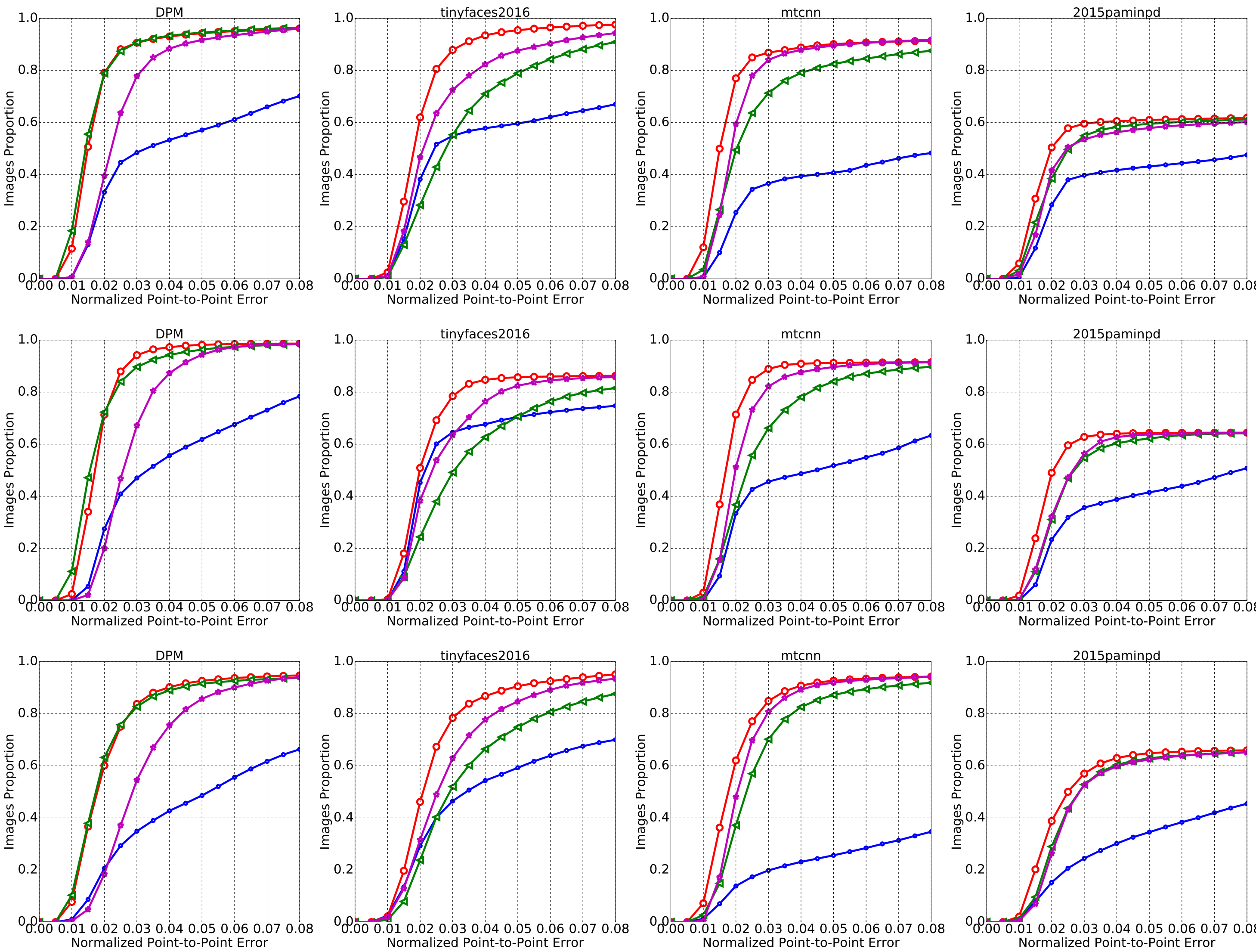

Fig. 3: This resembles the first set of curves as produced in the *Experiment 1* of the main paper. Rows are Category 1, 2 and 3 respectively. Each column is labelled by the figure header representing the work of [Felzenszwalb et al(2010)Felzenszwalb, Girshick, McAllester, and Ramanan], [Hu and Ramanan(2016)], [Zhang et al(2016)Zhang, Zhang, Li, and Qiao] and [Liao et al(2016)Liao, Jain, and Li] respectively.

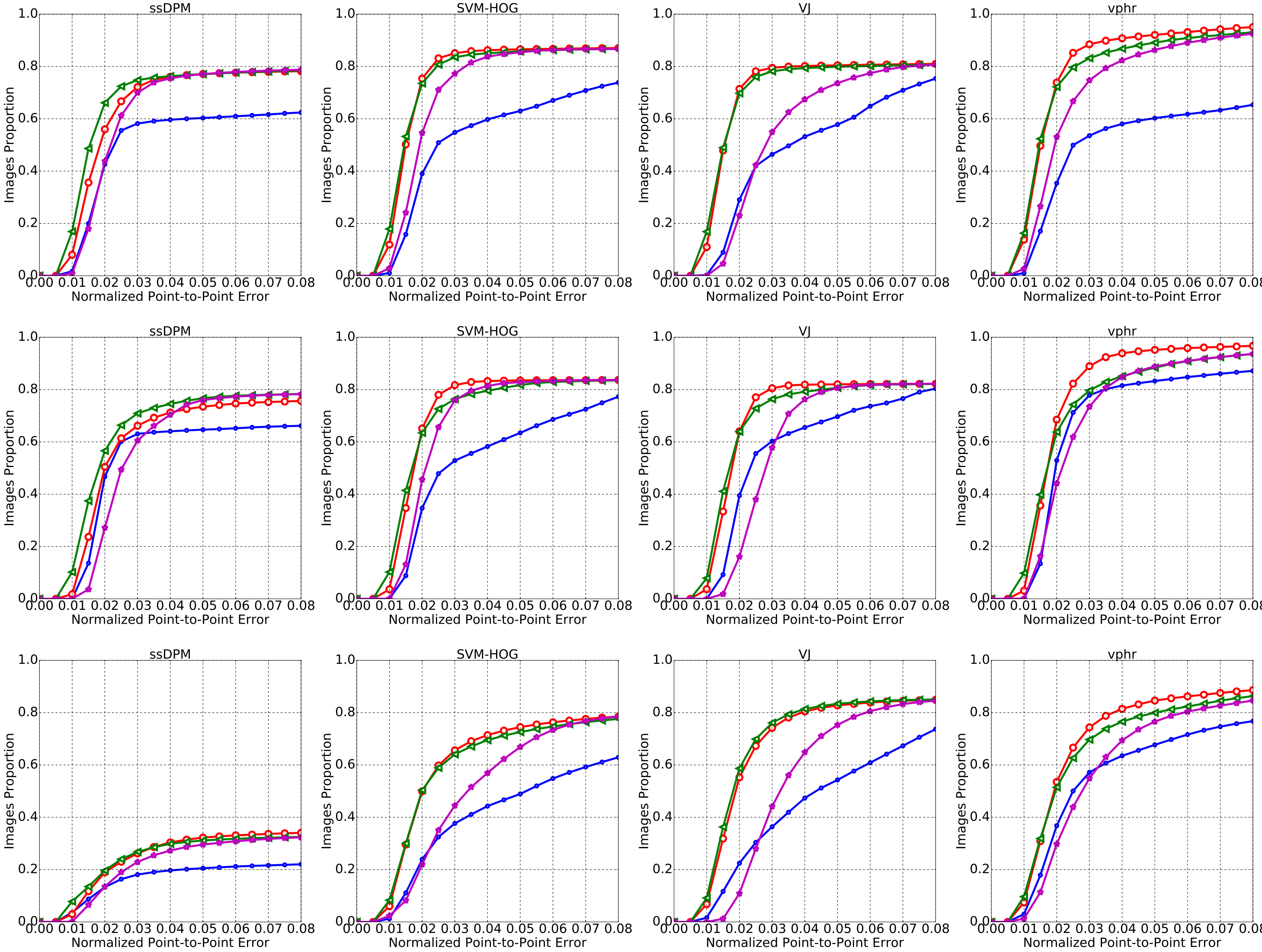

Fig. 4: This resembles the second set of curves as produced in the *Experiment 1* of the main paper. Rows are Category 1, 2 and 3 respectively. Each column is labelled by the figure header representing the work of [Zhu and Ramanan(2012)], [King(2015)], [Viola and Jones(2004)] and [Kumar et al(2015)Kumar, Namboodiri, and Jawahar] respectively.

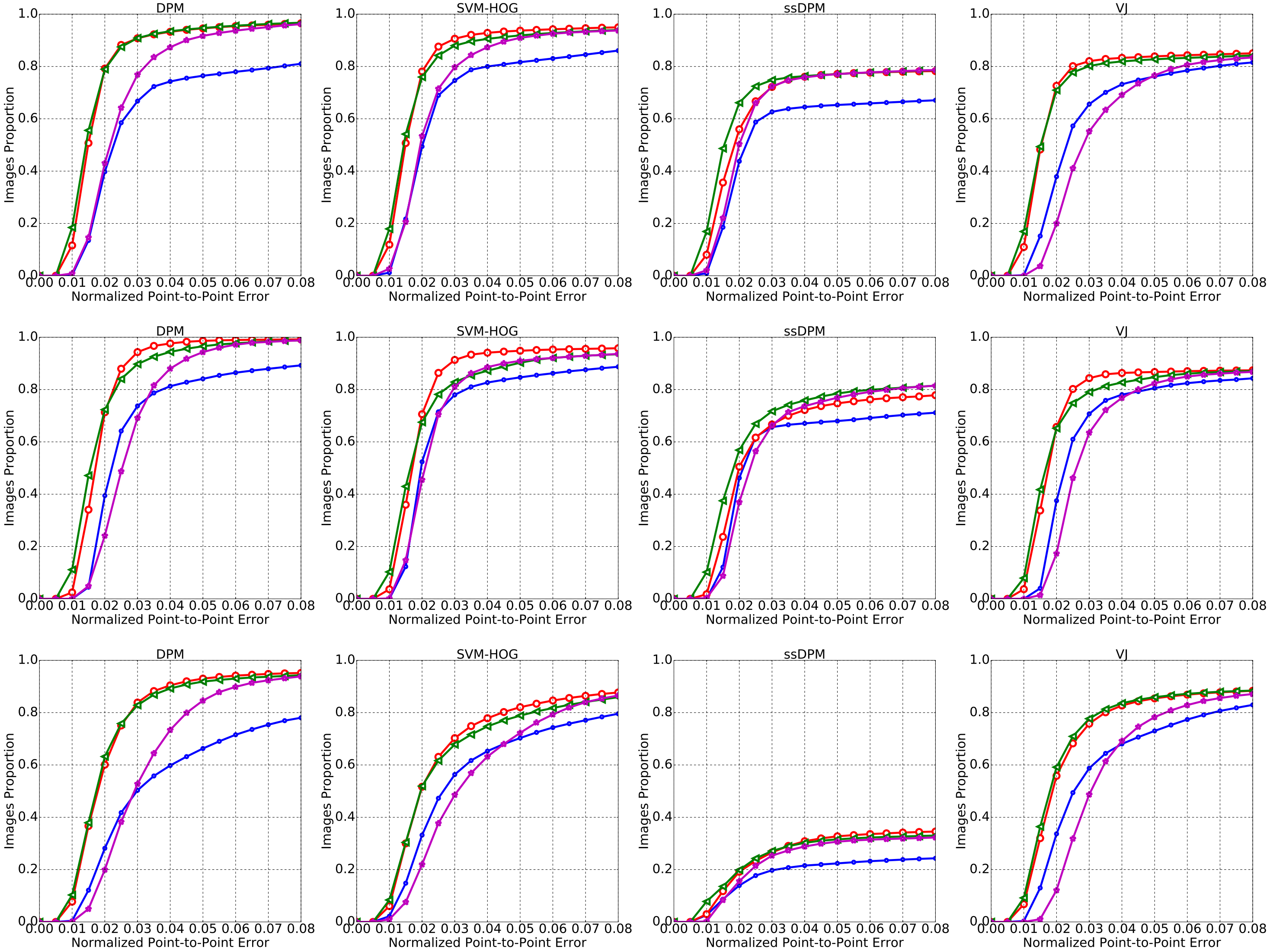

Fig. 5: This resembles the full set of curves as produced in the *Experiment 2* of the main paper. Rows are Category 1, 2 and 3 respectively. Each column is labelled by the figure header representing the work of [Felzenszwalb et al(2010)Felzenszwalb, Girshick, McAllester, and Ramanan], [King(2015)], [Zhu and Ramanan(2012)] and [Viola and Jones(2004)] respectively.

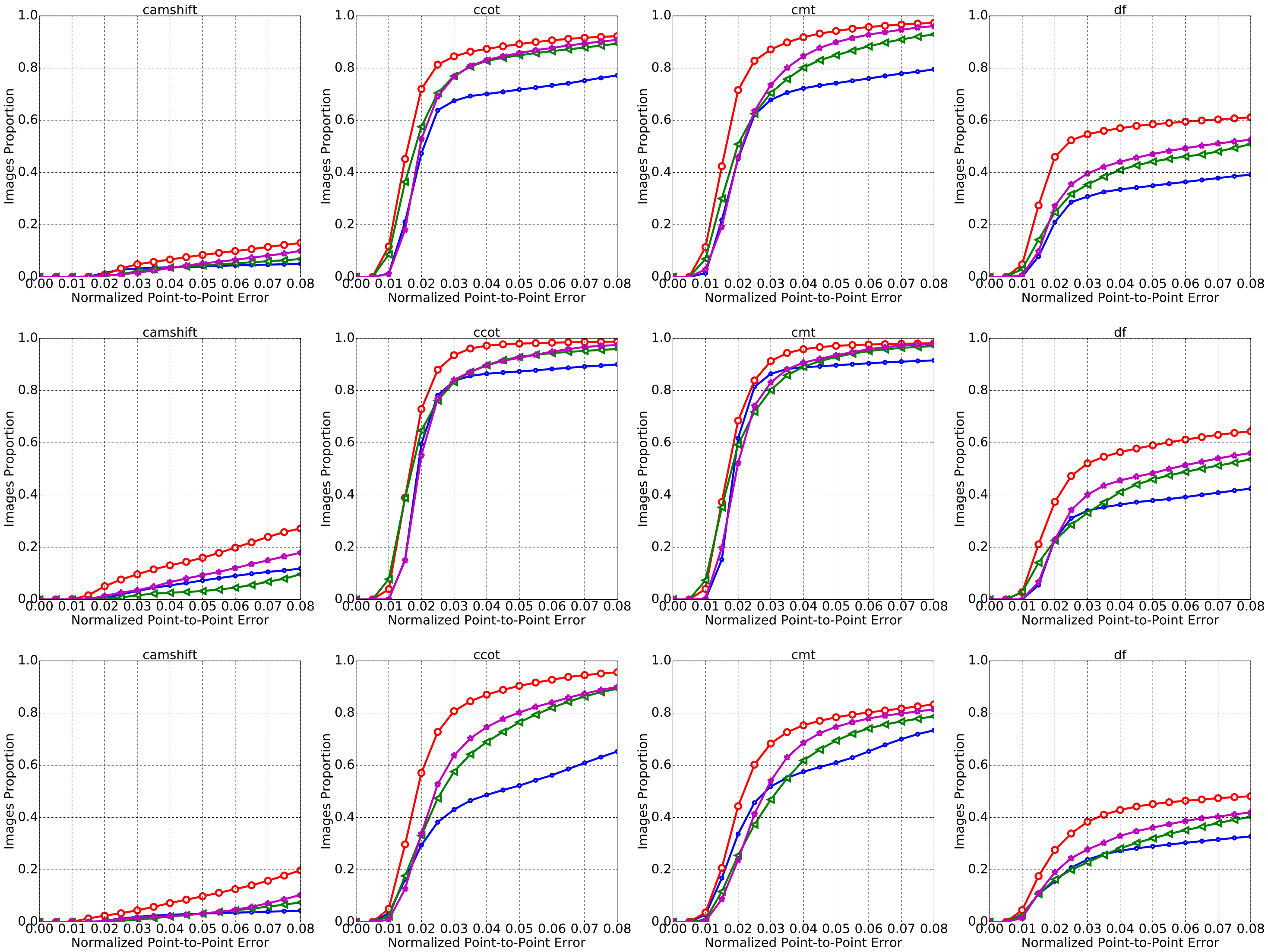

Fig. 6: This resembles the first part (out of 7) of the full set of curves as produced in the *Experiment 3* of the main paper. Rows are Category 1, 2 and 3 respectively. Each column is labelled by the figure header representing the work of [Bradski(1998)], [Danelljan et al(2016)Danelljan, Robinson, Khan, and Felsberg], [Nebehay and Pflugfelder(2015)] and [Sevilla-Lara and Learned-Miller(2012)] respectively.

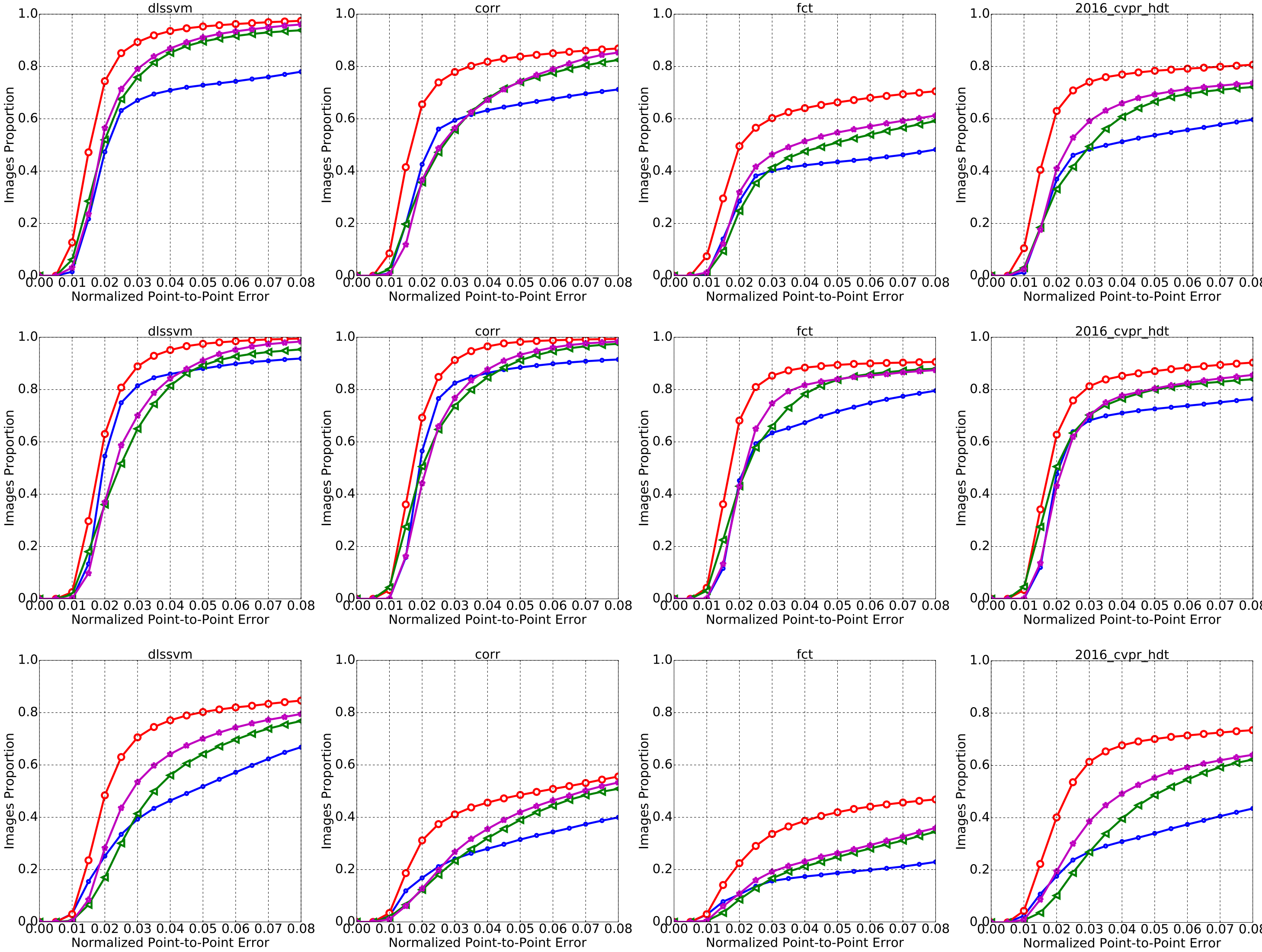

Fig. 7: This resembles the second part (out of 7) of the full set of curves as produced in the *Experiment 3* of the main paper. Rows are Category 1, 2 and 3 respectively. Each column is labelled by the figure header representing the work of [Sevilla-Lara and Learned-Miller(2012)], [Ning et al(2016)Ning, Yang, Jiang, Zhang, and Yang], [Danelljan et al(2014)Danelljan, Häger, Khan, and Felsberg] and [Zhang et al(2014c)Zhang, Zhang, and Yang] respectively.

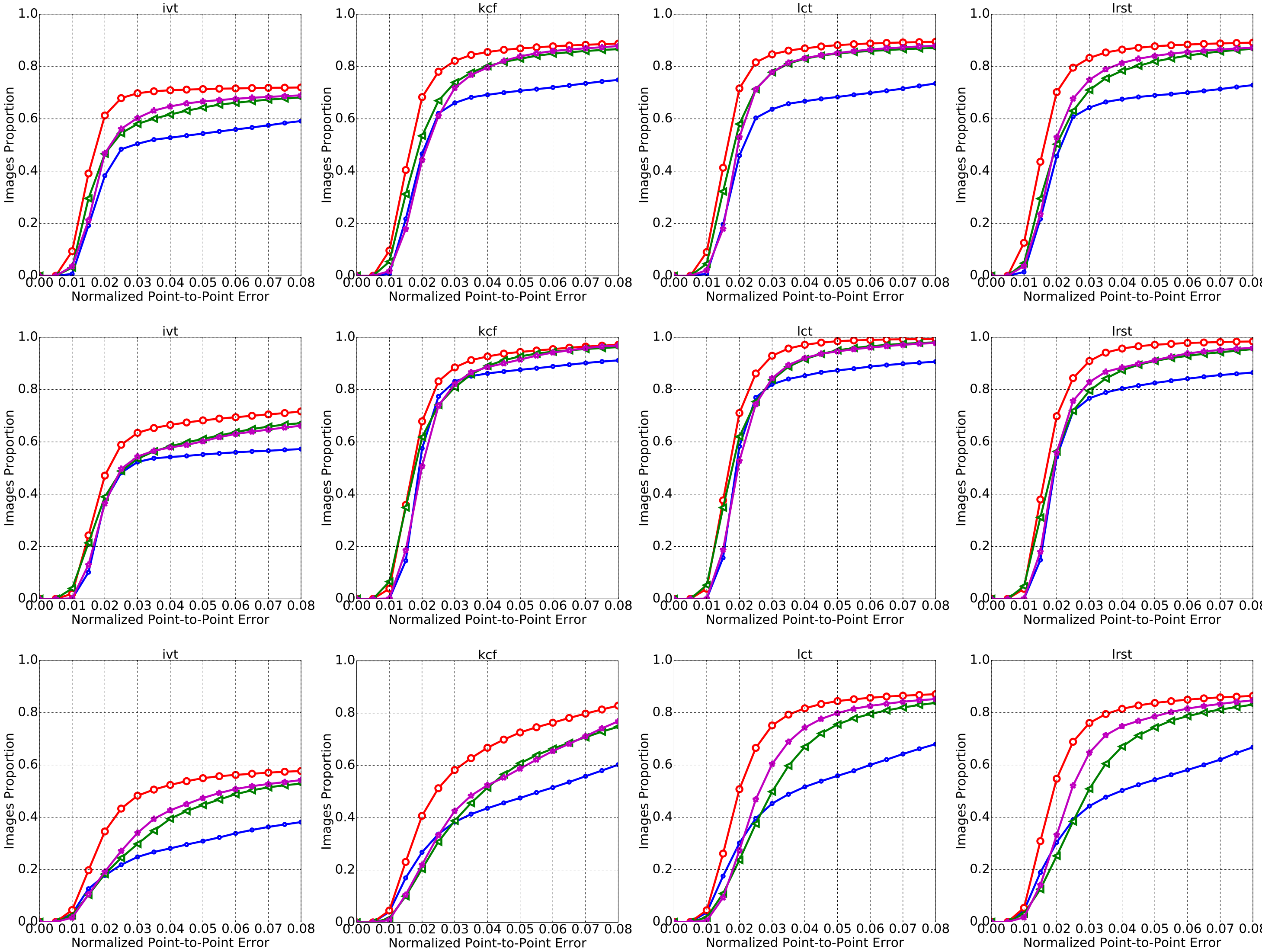

Fig. 8: This resembles the third part (out of 7) of the full set of curves as produced in the *Experiment 3* of the main paper. Rows are Category 1, 2 and 3 respectively. Each column is labelled by the figure header representing the work of [Ross et al(2008)Ross, Lim, Lin, and Yang], [Henriques et al(2015)Henriques, Caseiro, Martins, and Batista], [Ma et al(2015)Ma, Yang, Zhang, and Yang] and [Zhang et al(2014d)Zhang, Liu, Ahuja, Yang, and Ghanem] respectively.

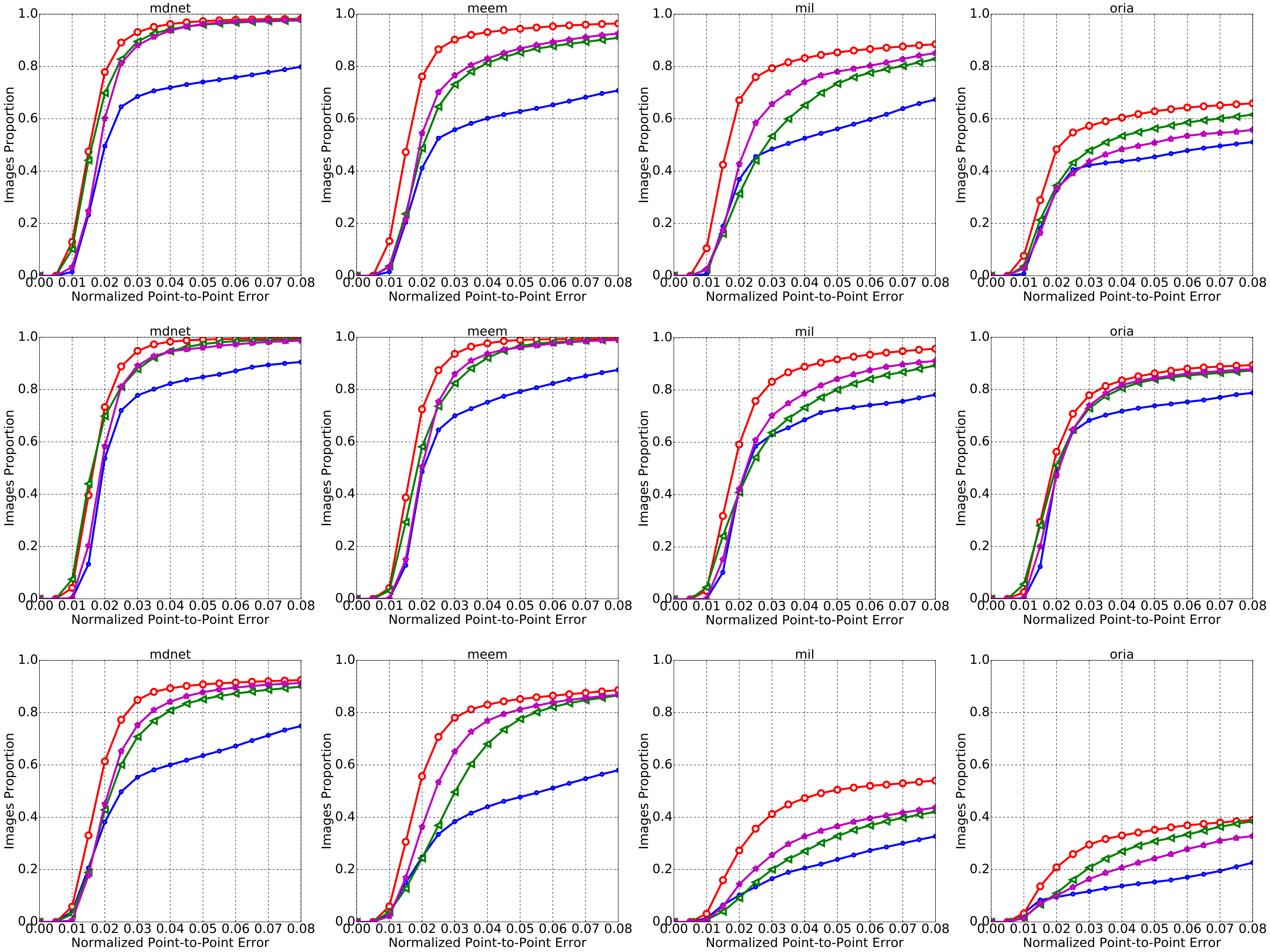

Fig. 9: This resembles the fourth part (out of 7) of the full set of curves as produced in the *Experiment 3* of the main paper. Rows are Category 1, 2 and 3 respectively. Each column is labelled by the figure header representing the work of [Nam and Han(2016)], [Zhang et al(2014a)Zhang, Ma, and Sclaroff], [Babenko et al(2011)Babenko, Yang, and Belongie] and [Wu et al(2012)Wu, Shen, and Ling] respectively.

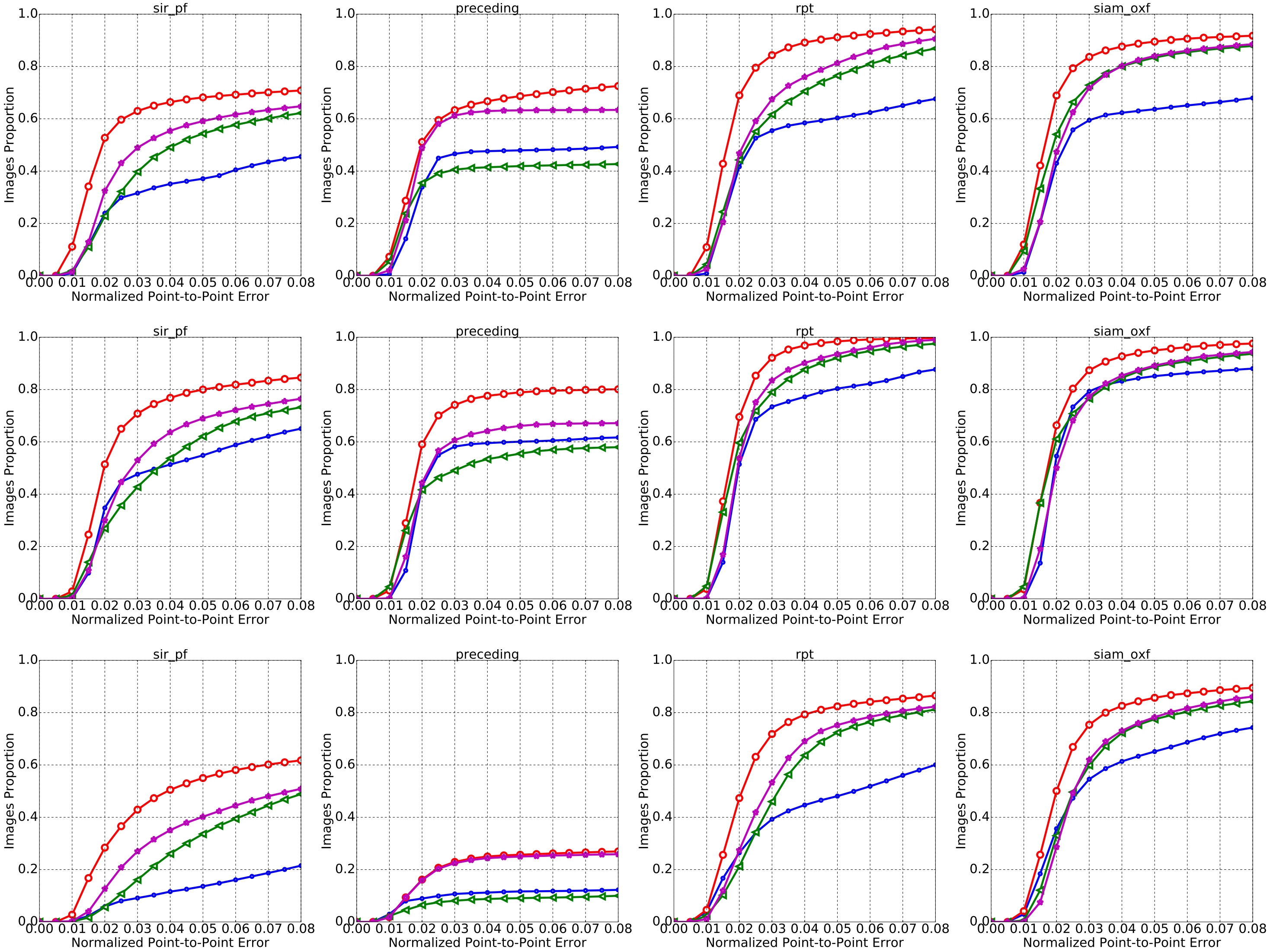

Fig. 10: This resembles the fifth part (out of 7) of the full set of curves as produced in the *Experiment 3* of the main paper. Rows are Category 1, 2 and 3 respectively. Each column is labelled by the figure header representing the work of [Isard and Blake(1996)], the baseline tracker (denoted as PREV in the main paper), [Li et al(2015)Li, Zhu, and Hoi] and [Bertinetto et al(2016b)Bertinetto, Valmadre, Henriques, Vedaldi, and Torr] respectively.

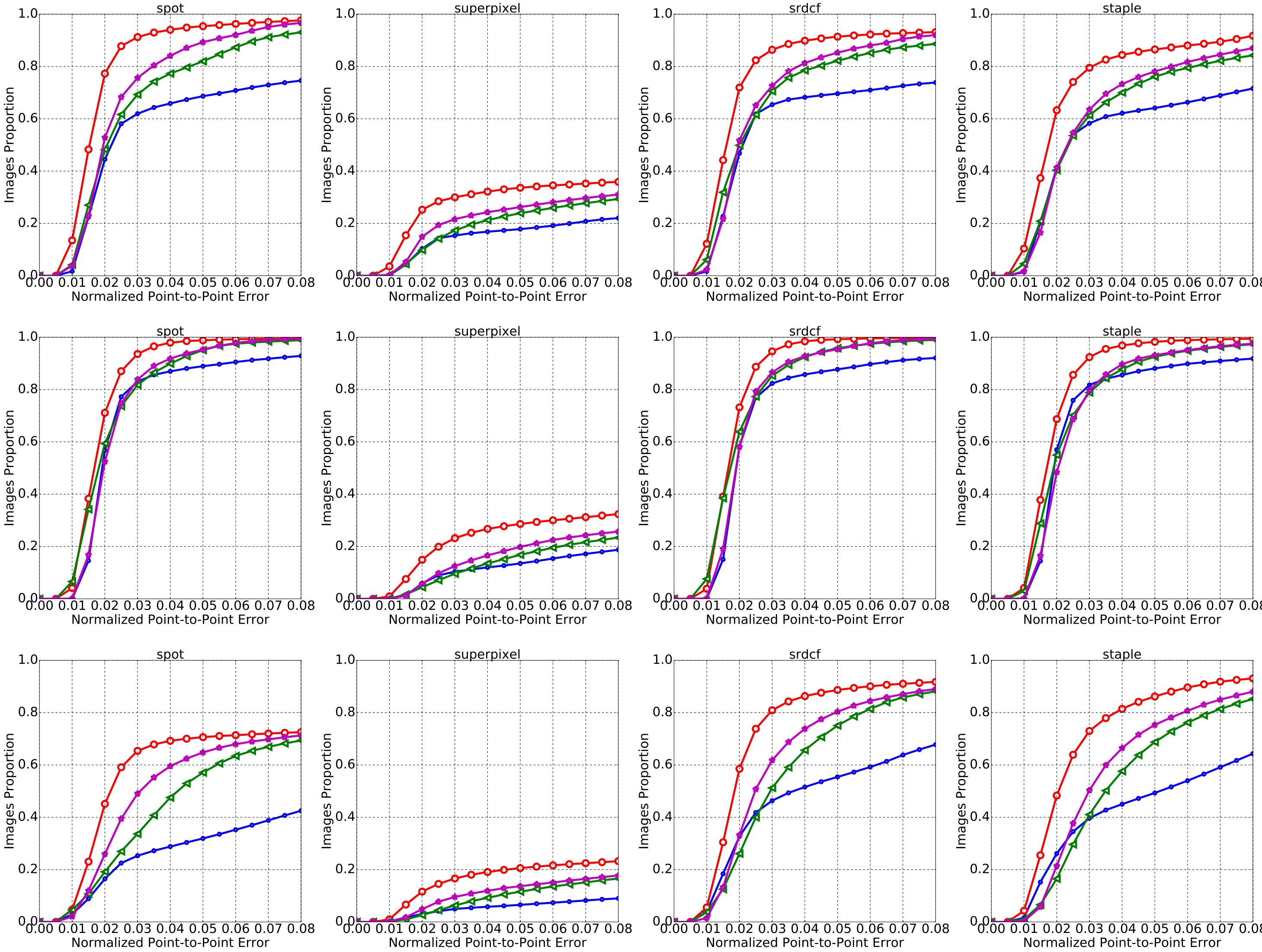

Fig. 11: This resembles the sixth part (out of 7) of the full set of curves as produced in the *Experiment 3* of the main paper. Rows are Category 1, 2 and 3 respectively. Each column is labelled by the figure header representing the work of [Zhang and van der Maaten(2014)], [Yang et al(2014)Yang, Lu, and Yang], [Danelljan et al(2015)Danelljan, Häger, Shahbaz Khan, and Felsberg] and [Bertinetto et al(2016a)Bertinetto, Valmadre, Golodetz, Miksik, and Torr] respectively.

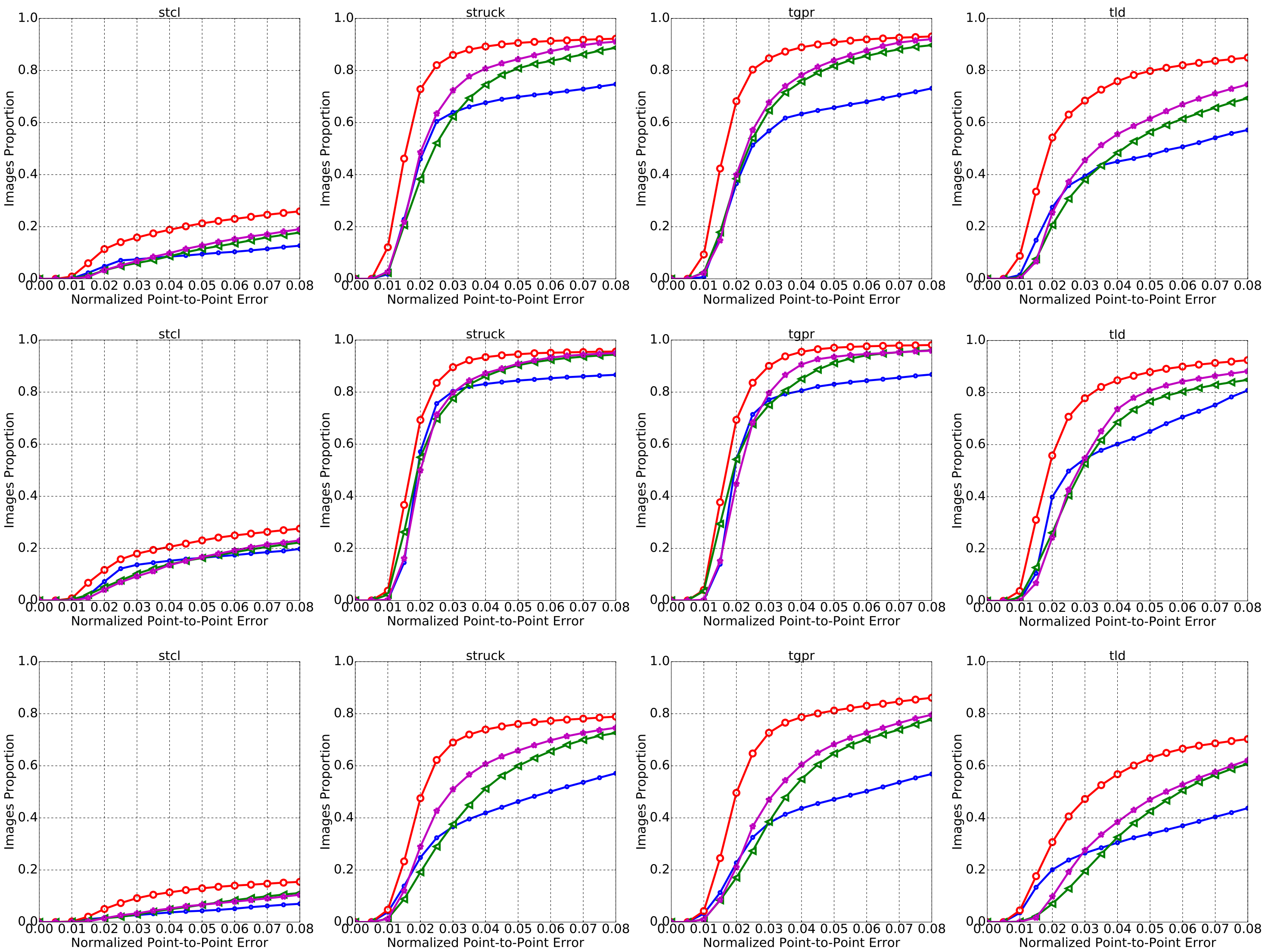

Fig. 12: This resembles the seventh part (out of 7) of the full set of curves as produced in the *Experiment 3* of the main paper. Rows are Category 1, 2 and 3 respectively. Each column is labelled by the figure header representing the work of [Zhang et al(2014b)Zhang, Zhang, Liu, Zhang, and Yang], [Hare et al(2011)Hare, Saffari, and Torr], [Gao et al(2014)Gao, Ling, Hu, and Xing] and [Kalal et al(2012)Kalal, Mikolajczyk, and Matas] respectively.

References

[Babenko et al(2011)Babenko, Yang, and Belongie] Babenko B, Yang MH, Belongie S (2011) Robust object tracking with online multiple instance learning. IEEE Transactions on Pattern Analysis and Machine Intelligence (T-PAMI) 33(8):1619–1632, DOI 10.1109/TPAMI.2010.226

[Bertinetto et al(2016a)Bertinetto, Valmadre, Golodetz, Miksik, and Torr] Bertinetto L, Valmadre J, Golodetz S, Miksik O, Torr PHS (2016a) Staple: Complementary learners for real-time tracking. In: IEEE Proceedings of International Conference on Computer Vision and Pattern Recognition (CVPR), IEEE, [Code: https://github.com/bertinetto/staple, Status: Online; accessed 18-August-2016]

[Bertinetto et al(2016b)Bertinetto, Valmadre, Henriques, Vedaldi, and Torr] Bertinetto L, Valmadre J, Henriques JF, Vedaldi A, Torr P (2016b) Fully-convolutional siamese networks for object tracking. arXiv preprint arXiv:160609549

[Bradski(1998)] Bradski GR (1998) Computer vision face tracking for use in a perceptual user interface

[Danelljan et al(2014)Danelljan, Häger, Khan, and Felsberg] Danelljan M, Häger G, Khan FS, Felsberg M (2014) Accurate scale estimation for robust visual tracking. In: Proceedings of British Machine Vision Conference (BMVC)

[Danelljan et al(2015)Danelljan, Häger, Shahbaz Khan, and Felsberg] Danelljan M, Häger G, Shahbaz Khan F, Felsberg M (2015) Learning spatially regularized correlation filters for visual tracking. In: IEEE Proceedings of International Conference on Computer Vision (ICCV), pp 4310–4318

[Danelljan et al(2016)Danelljan, Robinson, Khan, and Felsberg] Danelljan M, Robinson A, Khan FS, Felsberg M (2016) Beyond correlation filters: Learning continuous convolution operators for visual tracking. In: Proceedings of European Conference on Computer Vision (ECCV), pp 472–488, [Code:https://github.com/martin-danelljan/Continuous-ConvOp, Status: Online; accessed 22-December-2016]

[Felzenszwalb et al(2010)Felzenszwalb, Girshick, McAllester, and Ramanan] Felzenszwalb PF, Girshick RB, McAllester D, Ramanan D (2010) Object detection with discriminatively trained part-based models. IEEE Transactions on Pattern Analysis and Machine Intelligence (T-PAMI) 32(9):1627–1645

[Gao et al(2014)Gao, Ling, Hu, and Xing] Gao J, Ling H, Hu W, Xing J (2014) Transfer learning based visual tracking with gaussian processes regression. In: Proceedings of European Conference on Computer Vision (ECCV), Springer, pp 188–203, [Code: http://www.dabi.temple.edu/~hbling/code/TGPR.htm, Status: Online; accessed 4-December-2016]

[Hare et al(2011)Hare, Saffari, and Torr] Hare S, Saffari A, Torr PH (2011) Struck: Structured output tracking with kernels. In: IEEE Proceedings of International Conference on Computer Vision (ICCV), IEEE, pp 263–270

[Henriques et al(2015)Henriques, Caseiro, Martins, and Batista] Henriques JF, Caseiro R, Martins P, Batista J (2015) High-speed tracking with kernelized correlation filters. IEEE Transactions on Pattern Analysis and Machine Intelligence (T-PAMI) 37(3):583–596

[Hu and Ramanan(2016)] Hu P, Ramanan D (2016) Finding tiny faces. arXiv preprint arXiv:161204402 [Code: https://www.cs.cmu.edu/~peiyunh/tiny/, Status: Online; accessed 24-December-2016]

[Isard and Blake(1996)] Isard M, Blake A (1996) Contour tracking by stochastic propagation of conditional density. In: Proceedings of European Conference on Computer Vision (ECCV), pp 343–356, [Code: https://github.com/gnebehay/SIR-PF, Status: Online; accessed 23-December-2016]

[Kalal et al(2012)Kalal, Mikolajczyk, and Matas] Kalal Z, Mikolajczyk K, Matas J (2012) Tracking-learning-detection. IEEE Transactions on Pattern Analysis and Machine Intelligence (T-PAMI) 34(7):1409–1422

[King(2015)] King DE (2015) Max-margin object detection. arXiv preprint arXiv:150200046

[Kumar et al(2015)Kumar, Namboodiri, and Jawahar] Kumar V, Namboodiri A, Jawahar C (2015) Visual phrases for exemplar face detection. In: IEEE Proceedings of International Conference on Computer Vision (ICCV), pp 1994–2002, [Code: http://cvit.iiit.ac.in/projects/exemplar/, Status: Online; accessed 24-December-2016]

[Li et al(2015)Li, Zhu, and Hoi] Li Y, Zhu J, Hoi SC (2015) Reliable patch trackers: Robust visual tracking by exploiting reliable patches. In: IEEE Proceedings of International Conference on Computer Vision and Pattern Recognition (CVPR), pp 353–361

[Liao et al(2016)Liao, Jain, and Li] Liao S, Jain AK, Li SZ (2016) A fast and accurate unconstrained face detector. IEEE Transactions on Pattern Analysis and Machine Intelligence (T-PAMI) 38(2):211–223, [Code: http://www.cbsr.ia.ac.cn/users/scliao/projects/npdface/, Status: Online; accessed 24-December-2016]

[Ma et al(2015)Ma, Yang, Zhang, and Yang] Ma C, Yang X, Zhang C, Yang MH (2015) Long-term correlation tracking. In: IEEE Proceedings of International Conference on Computer Vision and Pattern Recognition (CVPR), IEEE, pp 5388–5396, [Code: https://github.com/chaoma99/lct-tracker, Status: Online; accessed 18-August-2016]

[Nam and Han(2016)] Nam H, Han B (2016) Learning multi-domain convolutional neural networks for visual tracking. In: IEEE Proceedings of International Conference on Computer Vision and Pattern Recognition (CVPR), IEEE, [Code: https://github.com/HyeonseobNam/MDNet, Status: Online; accessed 18-August-2016]

[Nebehay and Pflugfelder(2015)] Nebehay G, Pflugfelder R (2015) Clustering of Static-Adaptive correspondences for deformable object tracking. In: IEEE Proceedings of International Conference on Computer Vision and Pattern Recognition (CVPR), IEEE

[Ning et al(2016)Ning, Yang, Jiang, Zhang, and Yang] Ning J, Yang J, Jiang S, Zhang L, Yang MH (2016) Object tracking via dual linear structured svm and explicit feature map. In: IEEE Proceedings of International Conference on Computer Vision and Pattern Recognition (CVPR), [Code: www4.comp.polyu.edu.hk/~cslzhang/code/DLSSVM_CVPR.zip, Status: Online; accessed 18-August-2016]

[Ross et al(2008)Ross, Lim, Lin, and Yang] Ross DA, Lim J, Lin RS, Yang MH (2008) Incremental learning for robust visual tracking. International Journal of Computer Vision (IJCV) 77(1-3):125–141

[Sevilla-Lara and Learned-Miller(2012)] Sevilla-Lara L, Learned-Miller E (2012) Distribution fields for tracking. In: IEEE Proceedings of International Conference on Computer Vision and Pattern Recognition (CVPR), IEEE, pp 1910–1917

[Viola and Jones(2004)] Viola P, Jones MJ (2004) Robust real-time face detection. International Journal of Computer Vision (IJCV) 57(2):137–154

[Wu et al(2012)Wu, Shen, and Ling] Wu Y, Shen B, Ling H (2012) Online robust image alignment via iterative convex optimization. In: IEEE Proceedings of International Conference on Computer Vision and Pattern Recognition (CVPR), IEEE, pp 1808–1814

[Yang et al(2014)Yang, Lu, and Yang] Yang F, Lu H, Yang MH (2014) Robust superpixel tracking. IEEE Transactions in Image Processing (TIP) 23(4):1639–1651, [Code: `http://www.umiacs.umd.edu/~fyang/spt.html`, Status: Online; accessed 18-August-2016]

[Zhang et al(2014a)Zhang, Ma, and Sclaroff] Zhang J, Ma S, Sclaroff S (2014a) Meem: robust tracking via multiple experts using entropy minimization. In: Proceedings of European Conference on Computer Vision (ECCV), pp 188–203, [Code: `http://cs-people.bu.edu/jmzhang/MEEM/MEEM.html`, Status: Online; accessed 18-August-2016]

[Zhang et al(2014b)Zhang, Zhang, Liu, Zhang, and Yang] Zhang K, Zhang L, Liu Q, Zhang D, Yang MH (2014b) Fast visual tracking via dense spatio-temporal context learning. In: Proceedings of European Conference on Computer Vision (ECCV), pp 127–141, [Code: `http://www4.comp.polyu.edu.hk/~cslzhang/STC/STC.htm`, Status: Online; accessed 18-August-2016]

[Zhang et al(2014c)Zhang, Zhang, and Yang] Zhang K, Zhang L, Yang MH (2014c) Fast compressive tracking. IEEE Transactions on Pattern Analysis and Machine Intelligence (T-PAMI) 36(10):2002–2015

[Zhang et al(2016)Zhang, Zhang, Li, and Qiao] Zhang K, Zhang Z, Li Z, Qiao Y (2016) Joint face detection and alignment using multi-task cascaded convolutional networks 23(10):1499–1503, [Code: `https://github.com/kpzhang93/MTCNN_face_detection_alignment`, Status: Online; accessed 24-December-2016]

[Zhang and van der Maaten(2014)] Zhang L, van der Maaten L (2014) Preserving structure in model-free tracking. IEEE Transactions on Pattern Analysis and Machine Intelligence (T-PAMI) 36(4):756–769

[Zhang et al(2014d)Zhang, Liu, Ahuja, Yang, and Ghanem] Zhang T, Liu S, Ahuja N, Yang MH, Ghanem B (2014d) Robust visual tracking via consistent low-rank sparse learning. International Journal of Computer Vision (IJCV) 111(2):171–190

[Zhu and Ramanan(2012)] Zhu X, Ramanan D (2012) Face detection, pose estimation, and landmark localization in the wild. In: IEEE Proceedings of International Conference on Computer Vision and Pattern Recognition (CVPR), IEEE, pp 2879–2886
